# Sociometric network analysis in illicit drugs research: A scoping review

**DOI:** 10.1371/journal.pone.0282340

**Published:** 2023-02-27

**Authors:** Naomi Zakimi, Alissa Greer, Martin Bouchard, Arshpreet Dhillon, Alison Ritter

**Affiliations:** 1 School of Criminology, Simon Fraser University, Burnaby, British Columbia, Canada; 2 Drug Policy Modelling Program, Social Policy Research Centre, University of New South Wales, Sydney, New South Wales, Australia; Johns Hopkins University Bloomberg School of Public Health, UNITED STATES

## Abstract

**Background:**

Sociometric or whole network analysis, a method used to analyze relational patterns among social actors, emphasizes the role of social structure in shaping behaviour. Such method has been applied to many aspects of illicit drug research, including in the areas of public health, epidemiology, and criminology. Previous reviews about social networks and drugs have lacked a focus on the use of sociometric network analysis for illicit drugs research across disciplines. The current scoping review aimed to provide an overview of the sociometric network analysis methods used in illicit drugs research and to assess how such methods could be used for future research.

**Methods:**

A systematic search of six databases (Web of Science, ProQuest Sociology Collection, Political Science Complete, PubMed, Criminal Justice Abstracts, and PsycINFO) returned 72 relevant studies that met the inclusion criteria. To be included, studies had to mention illicit drugs and use whole social network analysis as one of their methods. Studies were summarized quantitatively and qualitatively using a data-charting form and a description of the studies’ main topics.

**Results:**

Sociometric network analysis in illicit drugs research has grown in popularity in the last decade, using mostly descriptive network metrics, such as degree centrality (72.2%) and density (44.4%). Studies were found to belong to three study domains. The first, drug crimes investigated network resilience and collaboration patterns in drug trafficking networks. The second domain, public health, focused on the social networks and social support of people who use drugs. Finally, the third domain focused on the collaboration networks of policy, law enforcement, and service providers.

**Conclusion:**

Future illicit drugs research using whole network SNA should include more diverse data sources and samples, incorporate mixed and qualitative methods, and apply social network analysis to study drug policy.

## Introduction

Social network analysis (SNA) is both a theoretical perspective and a methodological approach to examining the social connections and structures among social beings. Its foundations can be traced back to early 1900s business, anthropology, and sociology research [1–3]. Business and organizational research conducted at Harvard’s School of Business Administration played an important role in developing and popularizing early SNA methods [2, 4, 5]. Jacob Moreno and Helen Jennings, working within the psychiatric and psychological fields, are most often credited with the birth of SNA as we know it today [6–8]. Since then, SNA has been used in a wide variety of disciplines in the social sciences, such as anthropology, criminology, and education, and has turned into a “vibrant multidisciplinary field” [9]. SNA is a particularly useful approach because it treats people as interconnected social beings, emphasizing the role of structure in shaping behaviour. As a method, SNA provides a variety of tools stemming from graph theory to study all kinds of interactions, from human and animal relationships to institutional and government processes [2, 9].

Broadly speaking, social network studies can focus on a network as a whole (e.g., the network of all friendships in a classroom) or on egocentric networks (e.g., the individual network of each student) [10]. Most commonly, SNA studies will administer questionnaires and/or interviews to participants to elicit names of contacts that represent specific relationships or ties (i.e., social support, friendship) [11]. Another important type of network data comes from archival sources, which contain data that were not collected with the purpose of conducting SNA, such as police files or historical documents. This data collection strategy is useful when studying hard-to-reach populations, such as criminal organizations, politicians, or historical figures [12–14]. Although rare, observations can be a rich data source for researchers who are able to conduct fieldwork [15]. This data collection method can help uncover relationships participants may not have shared in a questionnaire or interview [16, 17]. Collectively, this variety of sources—observational, archival, and questionnaires or interviews—facilitates the multidisciplinary use of SNA.

Once data are collected, different analytical tools are available to study social networks. SNA techniques can be divided into three categories: descriptive network graphs, whole network or individual quantitative measures/metrics, and advanced network modelling [18]. First, descriptive network graphs can be used to visualize social ties. Network graphs can help illustrate and describe network data, as well as uncover relationship patterns that may be difficult to grasp using other methods. Second, network measures can be calculated for both the network as a whole or for individuals within the network. Whole network (or sociometric) measures—the interest for the current review—can provide information about density (i.e., the portion of total possible connections that actually exist in the network) and centralization (i.e., how focused a network is on a single node or person) [19, 20]. Individual-level measures can detect the most central member in a network in terms of how many connections they have (i.e., degree centrality) or how often they act as “brokers” connecting otherwise unconnected nodes (i.e., betweenness centrality) [19]. The last category of analysis includes more advanced network modelling techniques that can account for the autocorrelation that is present in network data, such as exponential random graph models (ERGM) [21]. Aside from these visual and quantitative measures, mixed methods that combine traditional SNA with qualitative data analysis can also be used to inform the SNA research design as well as to interpret findings [22, 23]. Using qualitative methods, such as thematic or discourse analysis, can provide context and meaning to quantitative SNA findings. While quantitative SNA can uncover relational patterns, qualitative methods can help interpret quantitative findings by answering how and why social connections form [23].

Needless to say, SNA can serve as a research toolbox with which to explore a wide range of social phenomena. This includes research on illicit drugs. The SNA approach is useful for the study of illicit drugs because it directly incorporates an important driver of drug market involvement—social relationships. For instance, drug use networks have structural characteristics that can be uncovered to aid in the understanding of HIV transmission. Risk behaviours may concentrate around core members who may later spread disease to those located in the periphery [24] and individuals who act as “brokers” (high in betweenness centrality) may be responsible for infecting other network members by bridging otherwise unconnected people [25]. Drug use networks may also have key individuals (articulation points) that connect people in the periphery of a network with harm reduction resources and information [26]. Drug markets can also be mapped using community detection methods to identify clusters of vendors and buyers that would otherwise go unnoticed [27]. In short, because drug use and drug policies affect and are affected by many aspects of society, such as public health [28], the environment [29], education [30], and the criminal justice system [31, 32], SNA can be a valuable approach to the study of these interconnected systems and the people within them. Such methods have already been applied to many aspects of illicit drugs research, including in the areas of public health, epidemiology, and criminology.

### Reviews on social network analysis in illicit drugs research

Thus far, reviews about illicit drugs and SNA have mapped out the literature within circumscribed disciplines (public health and criminology) and/or the focus has extended beyond illicit drugs to cover other types of drugs and populations. We identified seven published reviews of SNA and its application within illicit drugs research, in each case within a specific field or topic in public health or criminology. Three of the reviews focused exclusively on illicit drugs [33–35], while the rest studied other topics and populations in addition to illicit drug use or people who use drugs (e.g., tobacco use, sex workers, etc.). In the field of public health, three reviews narrowly focused on a variety of drug use and social network features among adolescents [36–38] and another three covered disease risk and transmission. For example, Jacobs et al. [37] analyzed the role of gender in adolescent use of alcohol, tobacco, and other drugs and found that sex composition in networks is rarely considered. In another systematic review, Henneberger et al. [36] focused on dynamic SNA, which captures changes over time, to understand peer selection and socialization in adolescents who use substances, noting that very few studies include adolescent drug use in their analyses. Similarly, Montgomery et al. [38] looked more broadly at studies about adolescent health behaviours, including illicit substance use, and found that adolescents tended to connect with peers that had similar health behaviours and that popularity, was associated with harmful health behaviours within adolescent social networks.

An additional three reviews focussed on the role of social networks in infectious disease prevention, risk, and/or transmission among people who use drugs. De et al. [33] analyzed 58 studies and reported that network structure and composition, as well as behavioural roles, can all impact drug equipment sharing among people who inject drugs. A more recent systematic review examined the literature on social support and HIV risk behaviours across different populations, including people who use drugs [39]. The authors did not find a consistent pattern across published studies in the association between social support and HIV risk behaviours in people who use drugs. Ghosh and colleagues [34] conducted a systematic scoping review of studies that used SNA and social network-based interventions to study HIV prevention and treatment in people who use drugs. The authors found that, in the area of HIV prevention and treatment, SNA had been mainly used as a secondary or exploratory method to uncover hidden populations, describe a network of relationships, or generate variables for further analysis.

In criminology, one systematic review published in 2017 contained 34 studies that used SNA to investigate organized crime groups involved in drug trafficking [35]. Among several key findings, SNA helped identify key individuals in a criminal network, and also showed that networks can adapt to increased law enforcement surveillance. While the use of SNA in criminology has arguably become “mainstream” [40], this is the only published review and assessment of existing literature on a topic related to drug crimes and the use of SNA.

In sum, these various reviews have lacked a focus on sociometric or whole network SNA in illicit drugs research across different disciplines. The current review aims to fill this gap by scoping the literature that uses SNA to study sociometric or whole networks (as opposed to ego or individual networks) related to illicit drugs to date. We focused on sociometric or whole network analysis because we were interested in understanding how SNA could be used to study social structures. While ego networks study the individual and their connections “in isolation from the structure of the network as a whole” [10], whole network analysis allows for the study of all connections within a bounded social sphere. Because the latter analysis focuses on the network in which individuals are embedded, data collection is often difficult: researchers must establish a network’s boundaries to identify all network members and their respective connections [41]. Whole network analysis can thus be extremely valuable in providing both a bird’s eye view of a social group, as well as individual perspectives, when researchers have access to the necessary data and sampling techniques. Drugs research spans a number of disciplines (eg., public health, economics, sociology, criminology). As such, there is a gap in the literature which examines the use of SNA across different disciplinary area. The study aims were to 1) provide an overview of whole network SNA methods used in illicit drugs research across disciplines, and 2) to describe the topics or study domains that have been covered to date using whole network methods. In addressing these study aims, we conclude by discussing the future of SNA in the field of illicit drugs research by identifying research gaps and opportunities for future studies.

## Methods

The current study followed Arksey and O’Malley [42] and Levac et al. [43] guidelines to conduct a scoping review, which consist of five required stages (described below) and an optional stakeholder consultation stage (the latter not employed here); further, to improve reporting quality, a PRISMA for Scoping Reviews Checklist (PRISMA-ScR) [44] is included in S1 Checklist. Whereas systematic reviews are ideal for collecting all existing literature on a topic and critically synthesizing results, often to inform practice, scoping reviews are useful tools when the main goal is to provide a rapid overview of a body of literature. Our aims were to provide a general overview of how sociometric or whole network SNA has been used in illicit drugs research across a variety of academic fields by describing the SNA methods used (in terms of data sources and collection tools, sample type, types of ties or connections among social actors, and analysis type), identifying the main topics in the literature, and discussing potential future research directions and gaps. A scoping review is therefore best aligned with the purposes of our study [45].

To find articles that were concerned with illicit drugs and referred to or used SNA, we searched six databases: Web of Science, ProQuest Sociology Collection, Political Science Complete, PubMed, Criminal Justice Abstracts, and PsycINFO. These databases were selected to encompass a variety of academic disciplines, given that SNA can be used as a method across the social sciences. The search process was iterative; different combinations of search terms were tried by two of the authors (NZ & AD) and later discussed by the entire research team (NZ, AD, MB, AR, AG). Ultimately, the following keywords were used to search for relevant titles, abstracts, and article keywords: (("illegal drug*") OR ("illicit drug*") OR ("illicit substance*") OR ("illegal substance*") OR (opioid*) OR (narcotic*) OR ("injection drug use") OR ("people who use drugs") OR ("drug user*") OR ("drug traffick*") OR ("drug deal*") OR ("drug* supply") OR ("drug* market") OR ("harm reduction") OR ("harm minimization") OR (overdos*) OR (“peer support”) OR (“peer worker*”) OR (“recovery peer*”)) AND (“network* analys*"). The final search strategy for Web of Science can be found in S1 Appendix. All searches were conducted between April 1^st^ and April 8^th^ 2022 by two research assistants (NZ and AD) who met regularly to discuss the search results. In total, the search returned 532 studies. After we removed duplicates, 237 unique studies remained and were saved in an Excel spreadsheet to assess whether they met the inclusion criteria.

All authors discussed inclusion and exclusion criteria several times. This approach was an iterative process [43]; inclusion and exclusion criteria changed and adapted to ensure the review would meet our research aims. No restrictions were placed on place of publication or publication status (government reports and theses were included). Further, because the current study focusses on whole network analyses across multiple disciplines we did not restrict publication date. To be included, studies had to be written in English, focus on illicit drugs, and use SNA as one of their methods. Studies that were about legal substances (alcohol, tobacco, e-cigarettes, prescription drugs, or other medications) (n = 37) or which were not about drugs at all were excluded (n = 11). Cannabis was considered an illicit substance due to still being illegal in most countries. Further, to be considered as SNA, studies had to have collected social network data systematically by establishing connections between individuals and/or organizations (i.e., asking participants whom they interacted with or coding interactions for people using court documents). Studies that mentioned networks without systematically collecting social network data were excluded (n = 3). This decision was based on the many ways in which the terms “social networks” can be used in the literature, but which do not strictly follow SNA data collection and analysis methods.

We also placed restrictions on the types of networks that could be included. First, studies about illicit drugs had to use sociometric or whole SNA as one of their methods [10]. Sociometric studies are ones that focus on the social structure of networks as a whole, such as capturing data on an entire drug trafficking organization or the social connections of a group of people who use drugs. Articles where SNA was used to study ego networks (i.e., networks of individual agents from their perspective as opposed to a whole network) were excluded (n = 21) [e.g., 46, 47]. Second, actors represented people or organizations, and the ties had to be social in nature (e.g., interactions, communication, criminal collaboration, sex, drug sharing, etc.). We excluded networks that did not meet these criteria (n = 74) (e.g., molecular networks, semantic networks, genetic networks, disease networks, brain networks, ecological networks, comorbidity networks, symptom networks, correlation networks, bibliography and co-citation networks, thematic networks, trafficking route networks). Finally, studies that used the same dataset in separate publications were treated as individual instances if the authors used the data in different analyses or to answer different research questions from those of previous studies.

**Fig 1:**
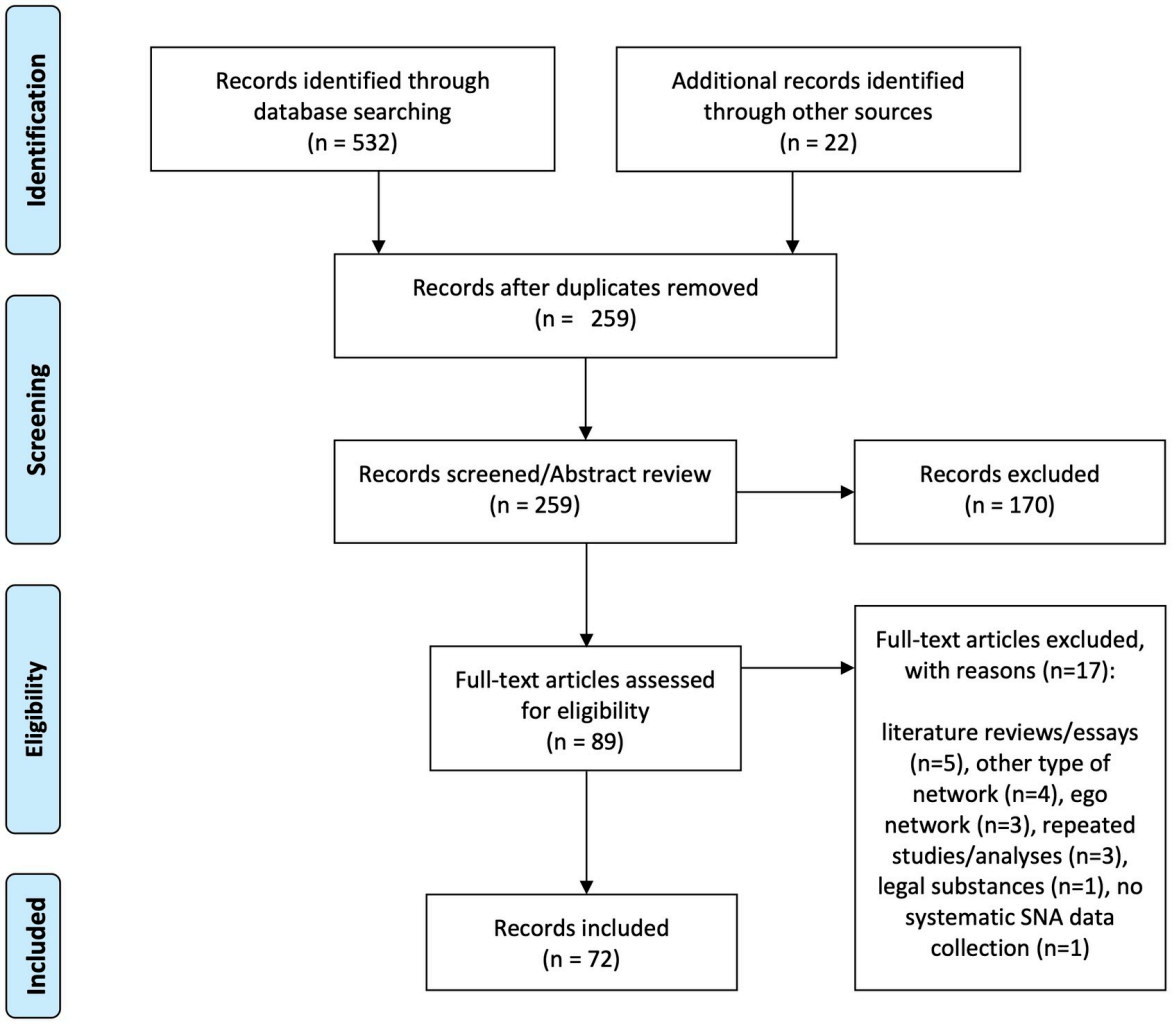
PRISMA 2009 flow diagram. Study selection process. *From*: Moher D, Liberati A, Tetzlaff J, Altman DG, The PRISMA Group (2009). *P*referred *R*eporting *I*tems for *S*ystematic Reviews and *M*eta-*A*nalyses: The PRISMA Statement. PLoS Med 6(7): e1000097. doi:10.1371/journal.pmed1000097 **For more information, visit** www.prisma-statement.org.

Following refinement of our search terms and strategy, the titles and abstracts of all 237 studies were reviewed against the inclusion criteria by two of the authors (AD and NZ) who regularly met to discuss their progress and resolve disagreements. We identified 50 studies as potentially relevant after the title and abstract review. An additional 22 studies were identified by reviewing the reference list of 10 randomly selected studies of similar published reviews [33, 35, 39], and by including articles already known to the authors, which included grey literature such as government reports and theses (no specific grey literature databases were used, however). In total, after a full-text review, 72 studies were included in the final body of texts for analysis (Fig 1).

A data-charting form was developed collaboratively by all authors to extract the content of the studies based on the study aims. Examples of data extracted included year of publication, data sources, sampling methods, and types of analyses. Three of the authors (AD, AG, and NZ) individually read and extracted information from five randomly selected studies and met twice to discuss the coding and revise the charting criteria. All authors discussed these changes and approved the subsequent charting form. Following Levac et al. [43], data were then extracted from all studies by two co-authors(NZ & AD) who met weekly to discuss their progress and any disagreements in coding. Whenever a disagreement occurred, the two authors discussed their differences and proposed solutions. For instance, the coders initially disagreed about how to code sampling techniques when no specific technique was mentioned. Upon discussion, the coders agreed that studies that did not name a particular sampling technique, such as snowball or purposive sampling, should be coded as “unspecified.” The codebook was then changed to the agreed upon definition and any previously coded data were revised to ensure the new changes were implemented. A third author (AG) was also consulted periodically to discuss major disagreements, if any, and resolve them. Through this process, the charting criteria continued to evolve to ensure the best available and relevant information was gathered. The list of included variables and the final data-charting form can be found in the S2 Appendix and S1 Dataset).

Our analysis of the studies’ methods and topics consisted of extracting both quantitative and qualitative patterns. We followed Levac et al.’s [43] advice to use a qualitative approach to develop categories to describe the studies reviewed. We collaboratively identified study domains using Excel and NVivo 12 [48] and conducted three rounds of coding to sort and analyze the data. The study domains were developed with the research aims in mind and followed a combination of deductive and inductive analysis. Two of the authors (NZ and AD) discussed and developed an initial list of three overarching study domains as they read and coded all the articles: criminal networks, networks of people who use drugs, and institutional networks. One researcher (NZ) then read the studies’ abstracts two times to refine the study domains or topic areas by creating subcategories for each domain. The study domains and their subcategories presented here are descriptive rather than interpretive, in line with the objectives of a scoping review.

In the findings section, we first present a descriptive quantitative summary of the studies’ characteristics and methods, followed by a narrative description of the main topics found in the studies. Last, we conclude by identifying research gaps and discussing the implications of our findings for future illicit drugs research.

## Findings

### Summary of studies

Table 1 presents a summary of all studies included in the scoping review. Year of publication ranged from 1997 to 2021, with a median of 2013, indicating that half of illicit drugs studies that use SNA were published in the past decade. Most studies’ samples were from the Global North or from multiple countries: 27 were from the U.S. (37.50%), 17 from multiple countries or online samples (23.61%), seven from Australia (9.72%), and six from Canada (8.33%).

**Table 1:** Summary of studies included in scoping review (n = 72).

	n	(%)
**Country of sample**		
US	27	37.50
Multiple countries/online sample	17	23.61
Australia	7	9.72
Canada	6	8.33
UK	2	2.78
Colombia	2	2.78
Turkey	2	2.78
Brazil	1	1.39
Lithuania	1	1.39
Iran	1	1.39
Serbia	1	1.39
Spain	1	1.39
Sweden	1	1.39
Greece	1	1.39
Italy	1	1.39
Uruguay	1	1.39
**Year of publication**		
Before 2000	3	4.17
2000–2010	18	20.83
2011–2020	45	62.50
2021 (Present)	6	12.50
**Data collection year**		
Before 2000	16	22.22
2000–2010	31	43.06
2011–2020	15	20.38
Unknown/incomplete	10	13.89
**Data sources**		
Primary	21	29.17
Archival/secondary	46	63.89
Both primary and archival	4	5.56
Unknown/incomplete	1	1.39
**Data collection tool** ^1^		
Prosecution/legal/police files	46	63.89
Questionnaire	19	26.39
Structured and semi-structured interviews	8	11.11
Websites/forums/social media	3	4.17
Media/books/news	6	8.33
Observations	4	5.56
Unknown/incomplete	1	1.39
**Reported sampling technique**		
Snowball or chain-referral sampling	16	22.22
*Respondent-driven sampling*	8	11.11
Purposive sampling	1	1.39
Specific technique unspecified/incomplete	55	76.39
**Sample**		
People/groups who sell/distribute/traffic drugs	44	61.11
People who use drugs and also sell drugs	2	2.78
People who use(d) drugs	20	27.78
Institutions/service providers/policymakers	6	8.33
**Datasets used in multiple studies**		
SNAP (Social Networks among Appalachian People)	5	6.94
Project Caviar	5	6.94
Operation Springtime	3	4.17
Operations Chalonero and Stupor Mundi	3	4.17
SFHR (Social Factors for HIV Risk)	2	2.78
Meth World	3	2.78
Natarajan (2000) cocaine trafficking organization	2	2.78
Natarajan (2006) heroin dealing organization	2	2.78
Turhal’s (2017) Turkish Anti-Smuggling and Organized Crime Department dataset	2	2.78
**Ties**		
Communication	19	26.39
Co-offending	20	27.78
*Drug transactions*	*4*	*5*.*56*
Social support, friendships, acquaintances	13	18.06
Collaboration or resource exchange	14	19.44
“Risky” relationship (drug partner and/or sex partner)	12	16.67
Other (peer education/advocacy, conflict, Twitter followers/retweets)	6	8.33
**Analysis type**		
**Quantitative inferential/predictive**	48	63.89
**Multivariable statistics**	17	22.22
**Bivariate statistics**	18	23.61
Other	12	16.67
Quantitative descriptive	15	20.83
Qualitative analysis	1	1.39
Both qualitative and quantitative methods^2^	8	13.89
Quantitative analysis complemented by description of qualitative data	4	
Mixed methods (qualitative analysis and quantitative analysis)	4	
**Relevant quantitative analytical methods** ^1^		
Network measures	67	93.06
Network visualizations	59	81.94
Community detection	14	19.44
ERGM	5	6.49
**Main network measures**		
Centrality measures		
Degree centrality	52	72.22
Betweenness centrality	39	54.17
Closeness centrality	12	16.67
Eigenvector centrality	9	12.50
Centralization measures		
Degree centralization	16	22.22
Betweenness centralization	6	8.33
Closeness centralization	3	4.17
Eigenvector centralization	1	1.39
Density	32	44.44
Community detection (cliques, core-periphery, components, k-cores, clustering coefficient)	34	47.22

^1^ Some studies used more than one data collection tool so the percentage for these categories may add up to more than 100%.

^2^ Studies that reported using qualitative analysis but did not specify how qualitative data were analyzed were coded as “quantitative analysis complemented by description of qualitative data”.

Studies’ data collection strategies were coded as either primary or archival/secondary. Studies that collected data by asking participants about their connections, such as in interviews or surveys, were coded as primary sources. Networks coded from pre-existing documents or data, such as court records, were coded as archival/secondary sources. As seen in Table 1, most of the studies used data collected from archival/secondary sources (n = 46; 63.9%) and the remaining studies used primary sources (n = 27; 29.2% except for four studies that combined both (5.56%). Specifically, network data were collected mainly from pre-existing official, legal, or government documents (n = 46; 63.9%) and questionnaires (n = 19; 26.4%). Some of the studies in our review used data from the same research project, such as SNAP (Social Networks among Appalachian People) (n = 5; 6.9%) and the Project Caviar dataset (n = 5; 6.9%). Researchers used these datasets to answer different research questions, and all were used for separate studies.

In most studies, a sampling technique was not specified (n = 55; 76.4%). These studies mostly used pre-existing documents to map full networks and, as such, simply included all actors in the documents as their sample. Studies that use archival data have little choice in terms of the sampling process, given that the data were collected for other purposes. Of the studies that mentioned a specific sampling technique, most used chain-referral or snowball methods (n = 16; 22.2%). Snowball or chain-referral sampling methods are particularly useful when attempting to map a network with unknown boundaries, where researchers do not known a priori who belongs to the group and who does not [41]. In such cases, people are recruited if they meet a set of criteria and they, in turn, refer other potential participants with whom they have social ties. This type of sample is non-random, but there are methods to make up for the lack of randomness. For example, respondent-driven sampling is a popular type of chain referral sampling that uses participant incentives to recruit unbiased samples Eight studies (11.1%) employed respondent-driven sampling, which is particularly well suited to uncover hidden networks [49]. In terms of the sampled populations, most studies sampled people or groups who sold, distributed, or trafficked drugs (n = 44; 61.1%) or people who use(d) drugs (n = 20; 27.8%).

The analytic approach for SNA was coded into four categories: quantitative inferential/predictive analysis, quantitative descriptive analysis, qualitative analysis, or a combination of both quantitative and qualitative methods. Most studies used quantitative inferential/predictive analyses (n = 46; 63.9%), which involved conducting a variety of statistical, mathematical, or computational analyses, from t-tests to exponential family random graph models (ERGM); half of these studies used bivariate inferential statistics (n = 17; 23.6%) (i.e., one dependent variable and one independent variable as opposed to multiple independent variables and at least one dependent variable). Fifteen studies used quantitative descriptive analyses (20.8%), which described network data using network graphs and/or variables (e.g., centralization) but did not conduct any statistical analysis. In terms of specific quantitative analytical methods, most studies reported at least one network measure, such as effective size, degree centrality, or betweenness centrality (n = 67; 93.1%). Beyond basic network measures, some studies also tested innovative algorithms or employed new quantitative methods to analyze SNA [50, 51].

Interestingly, eight studies reported using both quantitative and qualitative analysis to shed light on network dynamics that would be difficult to uncover from quantitative data alone. Four studies used qualitative data analysis like content or narrative analysis together with quantitative methods. However, the remaining four studies in this category lacked information on how qualitative data were analyzed; [52–55] instead, qualitative data like interviews and documents were used only descriptively to complement quantitative findings by citing interview excerpts or summarizing evidence from police files. Finally, one study used only qualitative analysis [56]; specifically, the authors qualitative content analysis to analyze data obtained through ethnographic observations, focus groups, and in-depth interviews.

Finally, Table 1 shows some of the most commonly used network measures (for more detail, the table in S1 Dataset contains specific information for all the network measures used by each study in the sample and S2 Appendix contains the definitions for each measure). Centrality measures such as degree, betweenness, closeness, and Eigenvector, are used to identify important or central individuals within a full network. Degree centrality, which is a simple measure of the number of individuals each network member is connected to, is the most commonly used in the current sample (n = 54). Betweenness centrality is also seemingly popular (n = 39); it identifies people who act as “bridges”, connecting otherwise unconnected network members. The other measures used in sociometric network studies are more focused on describing tendencies or patterns of the networks as a whole. Degree centralization, which measures how centralized a whole network is around one or few important individuals with many connections, is also frequently used (n = 16). However, other measures of network centralization, such as betweenness, closeness, and Eigenvector centralization, were rarely employed to analyze full networks in the current sample. Similarly, density and community measures were quite common. Density, which shows how many connections exist in a network out of all possible connections, were used in 32 studies. Finally, measures and algorithms can be used to identify communities or subgroups in a network, as reflected in 34 studies.

### Qualitative description of study domains

We identified and divided the studies reviewed into three domains of study: (1) the study of crime: drug trafficking or distribution networks, (2) public health and social support: the social networks of people who use drugs, and (3) policy, law enforcement, and service providers: institutional and policymaking networks. These study domains and their corresponding subcategories (see Table 2) present an overview of the topics covered in illicit drugs research that uses whole network SNA. Some studies belonged to more than one study domain. Similarly, most studies contained elements of several, if not all, subcategories within their respective domain to varying extents. Subcategories describe the topics covered in each domain and help explain how SNA has specifically been used for each topic.

**Table 2:** Summary of study domains, subcategories and corresponding studies.

STUDY DOMAIN	SUBCATEGORIES	STUDIES
1. The study of crime: drug trafficking or distribution networks	1.1 Resilience and disruption 1.2 Collaboration 1.3 Comparison of different types of networks	[27, 50, 51, 53–55, 57–98]
2. Public health and social support: the social networks of people who use drugs	2.1. Comparisons based on drug choice and drug use patterns 2.2 Disease risk and transmission	[24–26, 53, 56, 58, 91, 99–115]
3. Policy, law enforcement, and service providers: institutional and policymaking networks	-	[52, 53, 59, 68, 77, 106, 116, 117]

In Table 3, we also show how different networks measures were used across study domains. Studies that were categorized under the first study domain tended to use centrality measures (degree, betweenness, closeness, and eigenvector centrality) more than any other network measure (e.g., 98% used degree centrality). Study domain 1 contains studies about drug trafficking organizations, which often aim to identify key actors for network intervention or disruption by law enforcement. Studies about policy, law enforcement, and service providers in domain 3 also tended to favour centrality measures, especially degree (100%) and betweenness centrality (62.5%). Studies in domain 2, focused on public health and social support, were most likely to use degree centrality (50%) to identify important actors in a network. In all study domains, measures used to identify communities or subgroups were similarly common: about half of the studies in each domain employed these measures. Global measures such as density and centralization were not as common as centrality and community measures, with the exception of study domain 1 where half of the studies included density. Overall, degree centrality, density, and various community measures were the most commonly used across all study domains. Further, studies about crime and drugs were the most likely to use centralization measures to describe whole networks.

**Table 3:** Frequency of measures by study domain.

Network measure	Study domain 1: The study of crime (n = 49)	Study domain 2: Public health and social support (n = 24)	Study domain 3: Policy, law enforcement, and service providers (n = 8)
Degree centrality/average degree	48 (98.0%)	12 (50.0%)	8 (100%)
Betweenness centrality/average degree	36 (73.5%)	4 (16.7%)	5 (62.5%)
Closeness centrality/average closeness	10 (20.4%)	2 (8.3%)	2 (25.0%)
Eigenvector centrality/average eigenvector	7 (14.3%)	3 (12.5%)	1 (12.5%)
Degree centralization	14 (28.6%)	3 (12.5%)	1 (12.5%)
Betweenness centralization	5 (10.2%)	0 (0.0%)	0 (0.0%)
Closeness centralization	3 (6.1%)	0 (0.0%)	0 (0.0%)
Eigenvector centralization	1 (2.0%)	0 (0.0%)	0 (0.0%)
Density	24 (49.0%)	7 (29.2%)	0 (0.0%)
Community measures (cliques, core-periphery, components, k-cores, clustering coefficient)	23 (46.9%)	11 (45.8%)	5 (62.5%)

### Study domain 1

#### The study of crime: Drug trafficking or distribution networks

Broadly speaking, studies on drug crime networks shed light on the composition of networks involved in drug dealing or trafficking, both in terms of structural organization and individual roles within groups. Some studies focused on resilience and disruption and used SNA to assess how drug-related criminal networks, such as drug trafficking organizations or online drug markets, evolved over time, reacted to change, or identified points of disruption [e.g., 27, 61, 63, 73, 81, 86, 90, 118]. For example, Ünal [90] compared the structure of illicit drug trafficking networks against narco-terror networks using network measures to assess whether they prioritized a dense and efficient structure with visible central players or a more secure structure with short paths of information flow and small subgroups of trusted connections. Similarly, O’Reilly et al. [86] used longitudinal SNA to analyze the resilience and adaptability of a drug trafficking network in Australia over five time periods as they faced drug supply changes.

Studies in this study domain also highlighted the use of SNA in examining collaboration among people and groups committing drug-related crimes. Research on collaboration and co-offending highlight the social aspect of drug-related criminal networks. Visualizing a criminal group as a network, rather than as separate individuals, can allow researchers to understand the formation of criminal networks or organizations and help predict future intra- and inter-group conflict. Among studies reviewed, SNA was used to map networks of co-offending *within* drug markets and trafficking organizations [69, 70, 75], as well as to map interactions *between* groups or organizations [87, 88]. Looking at co-offending within groups, Heber [69] analyzed a network of co-offenders involved in drug crimes in Stockholm to investigate its structure, assess co-offending stability over time, and identify central members using descriptive network measures. At the between-group level, a doctoral dissertation presented an analysis of gang alliances and rivalries in the U.S., emphasizing the role of ethnicity, using open-source data, as well as descriptive network measures and community detection [87].

Identifying key players and central members was also an important part of understanding criminal networks and developing disruption strategies [e.g., 58, 61, 67]. Most of these studies proposed that using SNA can help agencies improve their targeting strategies, especially compared to policing methods not driven by data. Using data from criminal groups involved in cocaine trafficking, Gimenez-Salinas (2014) identified key players using degree and betweenness centrality and compared it to official police data. Basu and Sen [58] analyzed drug trafficking and terrorist groups to compare the traditional network approach of detecting key actors using centrality measures against a new proposed approach based on the mathematical concept of “identifying codes.” This model was developed to reduce the amount of resources needed to monitor network members compared to using standard centrality measures.

Overall, studies on drug-related criminal networks either aimed to understand how drug trafficking worked or to help law enforcement agencies improve their effectiveness in disrupting drug trafficking organizations and criminal groups. Both aims are not necessarily independent of each other: a better understanding of collaboration in drug trafficking networks may help identify points of vulnerability in these networks. The demonstration sometimes remains purely quantitative or in the realm of simulations; none of these articles, however, can truly answer questions on whether turning to SNA can be done while respecting criminal justice principles of fairness and proportionality. Yet, the potential of SNA to add new information on drug trafficking and distribution is relatively clear.

### Study domain 2

#### Public health and social support: The social networks of people who use drugs

The second study domain that we identified consists of whole network SNA research about people who use drugs and their social connections. Such studies were concerned with the effects of different types of social support or the role of peer influence (e.g., family, friendship, other people who use drugs) on specific behaviours or drug use patterns. For instance, Silva et al. [56] used network visualizations and qualitative content analysis to investigate the networks of social support for a sample of people who used crack cocaine and received support from a health program in Brazil. Another example is Arimoto [99], who examined the effects of peer influence on substance use—including alcohol, marijuana, and other “illegal/unauthorized” drugs like cocaine and methamphetamine—using ordered logistic regression but focused exclusively on adolescents. While all studies about the social networks of people who use drugs stressed the value of researching social support and peer influence using SNA at large, they also demonstrate how SNA can be used within three specific categories of interest: harm reduction diffusion, comparing social support and behaviour based on drug use patterns, and studying disease risk and transmission.

First, studies of networks about public health and social support illustrated the importance of social relationships on harm reduction behaviours or service access. These studies demonstrated how SNA can be used to study the diffusion of information and behaviour across a social network in a variety of contexts, as well as shed light on the importance of different social ties. Using SNA, Bouchard et al. [26] aimed to identify “harm reduction champions”—i.e., key network articulation points—who could effectively share harm reduction information across a network of people who use drugs. Along similar lines, Rudolph et al. [119] uncovered the minimum number of peer educators needed to reach at least half of the network of a sample of people who use drugs who visited high-risk sites. The authors used SNA and spatial data of people who use drugs to examine the association between overdosing or having connections to someone who had overdosed and individual and network level variables. Further, a study by Kwan et al. [120] looked at harm reduction services access; they assessed the importance of social relationships and methadone dosage on participation patterns in a low-threshold methadone treatment program in Hong Kong using bivariate statistics.

Second, a group of studies focused on comparing people’s social networks based on drugs used or drug use patterns. Overall, these studies showed how SNA can uncover the differential impact of certain drugs on an individual’s social relationships. Jonas et al. [104] compared the effective size of drug co-usage networks based on drug of choice (e.g., cannabis and OxyContin, among others) using multivariate statistical analysis. The network measure of effective size was used to measure social capital and identify differences based on drug of choice. Wendel et al. [91] used quantitative SNA, including ERGM, and software-assisted qualitative analysis of interview data to study methamphetamine users and sellers in New York City. The authors compared two submarkets, one composed of men who have sex with men and another one where methamphetamine use is not connected to participants’ sexual activity. Interestingly, one study investigated disinformation about cannabis and opioids (e.g., morphine, heroin, fentanyl, oxycodone, etc.) during the Covid-19 pandemic using Twitter data and descriptive network measures along with community detection techniques [115], drawing comparisons of network structural measures and disinformation between Twitter networks surrounding the two types of drug.

Third, a final category of studies about public health investigated disease transmission and risk behaviours. These studies show how SNA methods can be a valuable tool to predict and prevent disease transmission among people who use drugs. By analyzing the structure of social networks, researchers can identify individuals most likely to be at risk, as well as study the effectiveness of interventions designed to decrease risk behaviours. For example, Young et al. [114] created a network of risk relationships for people who use drugs who shared injection equipment and/or had unprotected sex and explored how receiving an HIV vaccine could result in an increase in risk behaviours using bivariate statistics. Gyarmathy et al. [25] studied how structural position (e.g., betweenness centrality, degree centrality) of people who injected drugs predicted HIV infection in a network of friends and family that provided advice and favours or with whom participants used drugs. In an interesting study about SNA methods, Bell et al. [100] used network simulations to assess whether collecting only ego network data about people who inject cocaine from the perspective of individuals could produce accurate results for the study of disease transmission compared to whole network data.

Studies in this domain shed light on how SNA can uncover the social aspect of drug use. Understanding group processes such as disease and information transmission within a network can be greatly facilitated by whole network SNA methods by allowing researchers to identify the most effective points of intervention within a group.

### Study domain 3

#### Policy, law enforcement, and service providers: Institutional and policymaking networks

Unlike the previous two study domains which captured the relationships of drug trafficking networks and people who use drugs, research within the third domain is about relationships among drug enforcement institutions, service providers, or policymakers. Studies such as the ones described in this section illustrate how SNA can help identify service gaps and collaboration relationships between service providers for people who use drugs. For instance, Spear [53] investigated a network of substance use treatment programs and the effects of these relationships on the likelihood of patient readmission using both bivariate and multivariable statistical analyses, as well as qualitative data description. Similarly, Murfree [106] researched the inter-organizational network of recovery service providers in Tennessee, U.S with community detection techniques and descriptive network measures.

Other studies were conducted on networks related to drug enforcement institutions, such as police and drug courts. These studies demonstrate the ways in which SNA to not only understand drug enforcement institutions and their collaboration patterns, but it may also be a valuable tool to hold government institutions accountable for their actions and uncover corruption. For example, Koturovic [116] built a “state response network” composed of institutions tasked with suppressing organized crime and drug trafficking in Serbia to identify structural holes and assess these organizations’ effectiveness in combatting crime using descriptive network measures and community detection techniques. Looking at Colombia and Mexico, Garay-Salamanca and Salcedo-Albarán [68] used traditional SNA methods coupled with an innovative method called SNAID (Social Network Analysis for Institutional Diagnosis) to explain how criminal networks can affect democratic formal institutions. Furthermore, two studies looked specifically at court processes related to illicit drugs [52, 77]. Looking at both individuals and organizations, Shomade [52] used quantitative analysis along with qualitative data description of one criminal and one drug court in the U.S. to shed light on the court structure and processes, as well as identify central members. Masias et al. [77] used machine learning techniques and SNA to predict the verdict in the trial of a drug trafficking organization based in Canada.

Finally, one study looked specifically at how drug policies were created. The study of policymaking using SNA can help governments, as well as advocacy groups and individuals, identify areas of improvement, such as ensuring all affected groups are represented in the process or that important individuals in the network are not isolated from the core. The only example of such a study in drug policymaking is a dissertation written by Musto [117]. The author dedicated a chapter to conducting SNA on the policymaking process that led to the creation of cannabis regulations in Uruguay between 2011 and 2013 using descriptive network measures. In this chapter, the author also used narrative analysis of qualitative interviews conducted in earlier sections to inform and interpret SNA findings.

Ultimately, it is clear that this study domain is less developed than others and presents many opportunities for future research, particularly using SNA to help inform more effective drug policies or regulations and to promote accountability.

## Discussion

The current scoping review sought to describe and examine how whole network or sociometric SNA methods have been used in illicit drugs research to reflect on and discuss the ways in which such methods can be used for future research. Overall, our quantitative summary shows that such studies have grown in popularity in the last ten years (2010–2021), are mostly from the U.S., and use quantitative methods. Upon a closer analysis of studies, our findings also show that whole network SNA has been used in three main study domains: drug crimes, public health and social support, and, to a lesser extent, policy, law enforcement and service providers. Specifically, most studies were concerned with uncovering the social dynamics of drug trafficking organizations and exploring how the social networks of people who use drugs promote and/or decrease risk behaviours and harm reduction opportunities.

This scoping review highlighted the strengths of whole network SNA in its ability to map complex behaviours and social relationships, across multiple domains, with both quantitative and qualitative data analytic techniques. First, future research could explore different sampling techniques and use more diverse data sources, especially in attempting to specify network boundaries. Second, qualitative and mixed methods should be implemented to provide more context on mechanisms operating within networks. Last, the third study domain could be expanded by studying drug policymaking networks and emphasizing the interconnectedness of all three study domains. We discuss each of these below.

First, it is important to consider the extent to which whole network studies truly and accurately represent a full network. Determining a network’s boundary—i.e., who belongs and who does not—is a key step in any whole network study that aims to map an entire group [41]. Ideally, networks should be mapped in their entirety when the population under study is known *a priori*; in this case, the boundaries are determined by the relationships or ties among the known members of the network. However, in practice, this is not always feasible, particularly when studying “hidden” or hard-to-reach populations, such as people who use drugs, drug trafficking groups, or policymakers. In these cases, boundaries may be difficult to establish, either because a group’s members are not publicly known, members may be reluctant to identify themselves as such and participate, or they may be hard to find. Snowball or chain-referral sampling methods are particularly useful when attempting to map a network with unknown boundaries [41], such as the ones used in a few of the studies in the current review. While there are several ways to approach sampling, network boundaries are likely to be constrained by the data sources available to researchers. In this sense, whole networks may not be necessarily “whole” and the resulting network measures may not be reliable if important data are missing or access to data is restricted.

Studies about criminal networks relied heavily on archival sources and studies about networks of people who use drugs tended to use questionnaires. Both types of data sources can be useful in uncovering networks of drug trafficking or people who use drugs; however, over-relying on a single data source may result in ignoring other types of networks or connections, leading to networks that may not be truly “whole” and may be missing key actors and ties. Future illicit drugs research could benefit from exploring the use of different data sources, such as autobiographies [121] or observations [52]. For example, studies about drug trafficking organizations could attempt to map full networks by interviewing known members to complement archival data provided by official government sources. We also found a lack of diversity in populations sampled: most studies used data from the Global North, echoing findings from previous systematic reviews by Bichler et al. [35] and De et al. [33], who found an overrepresentation of Western consumer countries with key positions in global trade and a lack of data on people who inject drugs from the Global South, respectively. Future illicit drugs research could explore social network structures and dynamics involved in drug use, drug crimes, drug enforcement, and drug policy in different countries with a variety of populations.

Second, our findings suggest that there is a lack of qualitative and mixed methods in whole network SNA research about illicit drugs across disciplines. This could be in part due to having included only studies that utilized systematic SNA data collection (i.e., asked specifically about social connections and did not use the concept as an analogy or metaphor), for which quantitative analysis is a better fit. However, illicit drugs researchers using quantitative methods could take advantage of and further refine the applicability of qualitative methods in quantitative SNA, which scholars have argued can provide important context and rich information [22, 122].

Last, the findings reveal that the third study domain remains largely unexplored: we found little SNA research about institutions, organizations, and individuals that create and enforce drug policies, especially as it concerns drug policymaking and inter-agency collaboration (e.g., between local and federal police, across different levels of government, etc.). Specifically, there was only one example of how whole network SNA methods can be used to study drug policymaking networks to date [117], but examples of SNA to study policymaking from other fields can serve as a starting point for the study of illicit drug policymaking. For example, policy network analysis has been used to study public health policymaking processes [123, 124], as well as environmental [125] and transport policies [126]. The use of SNA to study drug policymaking and institutional networks can reveal otherwise invisible dynamics about policymaking at different levels (e.g., individual and institutional), which can help create more effective and fair policies by increasing collaboration and identifying groups that are underrepresented in the policymaking process.

SNA research on drug policy is an important study domain on its own, but it must also be explored because of the major impact it can have on drug trafficking organizations (study domain #1) and public health (study domain #2). Given rapidly changing drug policies, such as the legalization of cannabis in countries like Canada and Uruguay [127, 128] and the decriminalization of drug possession in small amounts in British Columbia, Canada [129], the impact of such policy changes on organized crime and public health can be explored from a whole network SNA angle. For instance, drug policies aimed at decriminalizing and/or regulating or legalizing drugs may have an impact on the structure and behaviour of large criminal organizations involved in drug trafficking. Whole network SNA can help answer questions related to how organized groups evolve and adapt to new policies: do they decrease their involvement in drug trafficking, or do they turn their attention to new drug markets? Similarly, decriminalization and/or legalization of drugs and its impact on law enforcement practices will in turn have an effect on public health and on the lives of people who use drugs. Changes in social support networks and harm reduction efforts can be studied using whole network SNA by looking at affected communities before and after policy changes. If drug policies fail to meet the needs of affected communities, policymaking studies using SNA can also help drug policy advocates generate change by identifying strategic points of influence in a drug policy network.

The current scoping review is not without limitations. Given the capacity and aims of our project, studies about legal substances, such as alcohol, tobacco, and prescription drugs were excluded; however, we did note ample research using SNA in this area in our original literature search. A future scoping and/or systematic review should be conducted on these topics. Additionally, many types of networks, not just social ones, can be analyzed, including genetic, neural, and semantic networks—these were excluded from the current review. Thus, a thorough review of the use of SNA in other disciplines, such as environmental science, communications, urban studies, and public policy, could offer unique and innovative insights that the field of illicit drugs research could draw on. It is also important to note the limitations inherent to a scoping review. Scoping reviews are not as exhaustive as systematic reviews and do not aim to make quality assessments of the existing literature [45, 130]. Thus, our findings represent only an initial and possibly non-exhaustive attempt at mapping out the literature across different fields that use SNA to conduct illicit drugs research to draw insights on the potential of these methods and areas for future illicit drugs research.

We conclude with the suggestion that SNA may be well suited to studying illicit drugs from a whole-of-system approach, which takes into account how the health, social, and criminal justice systems overlap and how people are positioned within them [131]. A systems perspective is also compatible with a social determinants of health framework, which proposes that health is influenced by many different individual and system-level factors, such as people’s jobs, age, economic policies, and political regimes. These factors, in turn, can generate health inequities [132], as is the case for many people who use drugs who face different health barriers, such as structural racism, lack of social support, and lack of access to healthcare [133, 134]. Understanding all three study domains—people who use drugs, the criminal justice system, and drug policymaking—from a whole network perspective can help researchers visualize the different variables at all levels and across all systems that affect people who use drugs in specific contexts. No study thus far has taken this across-systems, multidisciplinary approach using whole network SNA methods in the area of illicit drugs research, creating a clear gap in the literature to address an issue as complex as illicit drugs.

## Supporting information

S1 Checklist Preferred Reporting Items for Systematic reviews and Meta-Analyses extension for Scoping Reviews (PRISMA-ScR) checklist. (PDF)

S1 Dataset Data-charting form. Data-charting form used to extract information from included studies (n = 72).
(XLSX)

S1 Appendix Search strategy for Web of Science. (PDF)

S2 Appendix List of variables. Variables included in the data-charting form and their definitions.
(PDF)

S3 Appendix Studies included in the scoping review (n = 72). (DOCX)
