# Supplementary material for: Sociometric network analysis in illicit drugs research: A scoping review
Source: PLoS One. 2023 Feb 27;18(2):e0282340. doi: 10.1371/journal.pone.0282340 (PMC9970099; doi:10.1371/journal.pone.0282340)
Supplement: S1 Appendix — (PDF) [file pone.0282340.s003.pdf]

S2 Appendix. Search strategy for Web of Science.

(TI=(("illegal drug*") OR ("illicit drug*") OR ("illicit substance*") OR ("illegal substance*") OR (opioid*) OR (narcotic*) OR ("injection drug use") OR ("people who use drugs") OR ("drug user*") OR ("drug traffick*") OR ("drug deal*") OR ("drug* supply") OR ("drug* market") OR ("harm reduction") OR ("harm minimization") OR (overdos*) OR (“peer support”) OR (“peer worker*”) OR (“recovery peer*”)) OR AB=(("illegal drug*") OR ("illicit drug*") OR ("illicit substance*") OR ("illegal substance*") OR (opioid*) OR (narcotic*) OR ("injection drug use") OR ("people who use drugs") OR ("drug user*") OR ("drug traffick*") OR ("drug deal*") OR ("drug* supply") OR ("drug* market") OR ("harm reduction") OR ("harm minimization") OR (overdos*) OR (“peer support”) OR (“peer worker*”) OR (“recovery peer*”)) OR AK=(("illegal drug*") OR ("illicit drug*") OR ("illicit substance*") OR ("illegal substance*") OR (opioid*) OR (narcotic*) OR ("injection drug use") OR ("people who use drugs") OR ("drug user*") OR ("drug traffick*") OR ("drug deal*") OR ("drug* supply") OR ("drug* market") OR ("harm reduction") OR ("harm minimization") OR (overdos*) OR (“peer support”) OR (“peer worker*”) OR (“recovery peer*”))) AND (TI=("network analys*") OR AB=("network analys*") OR AK=("network analys*"))
