# Supplementary material for: Sociometric network analysis in illicit drugs research: A scoping review
Source: PLoS One. 2023 Feb 27;18(2):e0282340. doi: 10.1371/journal.pone.0282340 (PMC9970099; doi:10.1371/journal.pone.0282340)
Supplement: S2 Appendix — Variables included in the data-charting form and their definitions. (PDF) [file pone.0282340.s004.pdf]

S3 Appendix. List of variables. Variables included in the data-charting form and their definitions.

Variable	Definition
**Dataset**	
**Authors**	Author(s) last names and initials.
**Year**	Year of publication.
**Sample Country**	Country participants were sampled from.
**Data Collection Year**	Year(s) when data were collected.
**Data Source**	Primary = Data collected for network analysis by asking people about their connections (e.g., survey, interview).<br>Archival/secondary = Data collected from existing document/video/transcripts originally created for purposes other than SNA (e.g., police files, court transcripts).<br>Both primary and archival/secondary = Combination of primary and secondary data.<br><br>If the study does not mention the data source, it was coded as unknown/incomplete.
**Data Collection Tool**	Tool used to collected network data, such as questionnaire, prosecution/legal/police files, media/books/news, observations, interviews. If more than one data collection tool was used, the word "multiple" was included at the end of the code.
**Sample**	Population sampled, including people who sell/distribute/traffic drugs, people who use drugs, institutions/service providers and policy actors.
**Sampling Technique**	Sampling technique if reported by the study's author(s), including snowball or chain-referral sampling, RDS (Respondent Driven Sampling), and purposive. If the sampling technique was not specified, the study was coded as "unspecified." If there were not enough information about the sampling methods, the study was coded as "incomplete."
**Ties**	Type of ties included in the social network, including friendship, social support, co-offending, drug co-usage, etc.
**Analysis Type**	Qualitative analysis: uses qualitative analysis to explain the network (e.g., interviews, thematic analysis of text, etc.).<br>Qualitative Descriptive: studies where qualitative data like interviews and documents are used only descriptively to complement quantitative findings (including quotes, providing context, etc.), but they lack information on how qualitative data were analyzed.<br>Quantitative descriptive: network data are systematically collected and used to describe the network. Network may have been visualized. Network measures may be used to describe the network.<br>Quantitative inferential/predictive analysis: uses quantitative statistical analysis to analyze network data (e.g., community detection, t-tests, ANOVA, regression, ERGMs, OLS, etc.).<br><br>A study may contain both quantitative and qualitative methods.
**Specific Analysis Type**	Specific analyses conducted, such as network visualizations, network measures (e.g., betweenness centrality), community detection, thematic analysis, etc.

**Type of Inferential/Predictive Analysis**	If the study used quantitative inferential/predictive analysis, this code specifies whether it used multivariable statistics, bivariate statistics, or other (e.g., machine learning). If a study conducted both multivariable and bivariate statistics, it was coded as multivariable.
**Main Network Measures**	Indicate any network measures used in the study, including but not limited to:<br>Degree centrality: measures the number of connections each actor has in a network.<br>Betweenness centrality: measures how often an actor is on the shortest path between two nodes in a network, connecting or "bridging" actors.<br>Closeness centrality: measures how far, on average, each actor is from other actors in the network<br>Eigenvector centrality: measures the importance of each actor's connections; connections to actors with higher centrality are weighted higher than connections to actors with few connections.<br>Degree centralization: measures the difference in degree centrality among nodes in the network; high scores indicate a network centers around one (or a few) individual(s) with high degree centrality.<br>Betweenness centralization: measures the difference in betweenness centrality among nodes in the network; high scores indicate a network centers around one (or a few) individual(s) with high betweenness centrality.<br>Closeness centralization: measures the difference in closeness centrality among nodes in the network; high scores indicate a network centers around one (or a few) individual(s) with high closeness centrality.<br>Eigenvector centralization: measures the difference in eigenvector centrality among nodes in the network; high scores indicate a network centers around one (or a few) individual(s) with high eigenvector centrality.<br>Density: measures how many ties or connections exist in the network out of all possible ties that could be formed.<br>Community measures: measures such as cliques, core-periphery, components, k-cores, and clustering coefficient help identify subgroups within the network.<br><br>Knoke D, Yang S. Social Network Analysis. Vol. 154, Social Network Analysis. Thousand Oaks: SAGE Publications, Inc; 2020. 1 p.
