# Supplementary material for: Sociometric network analysis in illicit drugs research: A scoping review
Source: PLoS One. 2023 Feb 27;18(2):e0282340. doi: 10.1371/journal.pone.0282340 (PMC9970099; doi:10.1371/journal.pone.0282340)
Supplement: S3 Appendix — (DOCX) [file pone.0282340.s005.docx]

**S5 Appendix. Studies included in the scoping review (n=72).**

1. Baika L, Campana P. Centrality, mobility, and specialization: a study of drug markets in a non-metropolitan area in the United Kingdom. Journal of Drug Issues. 2020;50(2):107-26.

2. Basu K, Sen A. Identifying individuals associated with organized criminal networks: a social network analysis. Social Networks. 2021;64:42-54.

3. Benítez GJ. Mapping Colombia's Counternarcotics Networks: The Rise of Latin American and Caribbean Partnerships. The Latin Americanist. 2019;63(3):275-306.

4. Bright DA, Greenhill C, Ritter A, Morselli C. Networks within networks: using multiple link types to examine network structure and identify key actors in a drug trafficking operation. Global Crime. 2015;16(3):219-37. doi: 10.1080/17440572.2015.1039164.

5. Bright DA, Greenhill C, Reynolds M, Ritter A, Morselli C. The Use of Actor-Level Attributes and Centrality Measures to Identify Key Actors: A Case Study of an Australian Drug Trafficking Network. Journal of Contemporary Criminal Justice. 2015;31(3):262-78. doi: 10.1177/1043986214553378.

6. Bright D, Hughes C, Chalmers J. Illuminating dark networks: a social network analysis of an Australian drug trafficking syndicate. Crime, Law & Social Change. 2012;57(2):151-76. doi: 10.1007/s10611-011-9336-z. PubMed PMID: 71346114.

7. Bright DA, Delaney JJ. Evolution of a drug trafficking network: Mapping changes in network structure and function across time. Global Crime. 2013;14(2-3):238-60. doi: 10.1080/17440572.2013.787927.

8. Calderoni F. The structure of drug trafficking mafias: the ‘Ndrangheta and cocaine. Crime, Law and Social Change. 2012;58(3):321-49. doi: 10.1007/s10611-012-9387-9.

9. Calderoni F, Skillicorn DB, Zheng Q. Inductive discovery of criminal group structure using spectral embedding. Information & Security: An International Journal. 2014;31:49-66.

10. Calderoni F. Strategic positioning in mafia networks. Crime and Networks: Routledge; 2013. p. 163-81.

11. Canter D. A partial order scalogram analysis of criminal network structures. Behaviormetrika. 2004;31(2):131-52.

12. Duxbury SW, Haynie DL. The network structure of opioid distribution on a darknet cryptomarket. Journal of Quantitative Criminology. 2018;34(4):921-41.

13. Gimenez-Salinas Framis A. Illegal networks or criminal organizations: Power, roles and facilitators in four cocaine trafficking structures. In: Morselli C, editor. Crime and Networks. New York: Routledge; 2013. p. 131-48.

14. Garay-Salamanca LJ, Salcedo-Albarán E. Institutional impact of criminal networks in Colombia and Mexico. Crime, Law and Social Change. 2011;57(2):177-94. doi: 10.1007/s10611-011-9338-x.

15. Heber A. The networks of drug offenders. Trends in Organized Crime. 2009;12(1):1-20. doi: 10.1007/s12117-008-9055-8.

16. Hofmann DC, Gallupe O. Leadership protection in drug-trafficking networks. Global Crime. 2015;16(2):123-38. doi: 10.1080/17440572.2015.1008627.

17. Hu D, Kaza S, Chen H. Identifying significant facilitators of dark network evolution. Journal of the American Society for Information Science and Technology. 2009;60(4):655-65. doi: 10.1002/asi.21008.

18. Hughes CE, Bright DA, Chalmers J. Social network analysis of Australian poly-drug trafficking networks: How do drug traffickers manage multiple illicit drugs? Social Networks. 2017;51:135-47. doi: 10.1016/j.socnet.2016.11.004.

19. Jones NP, Dittmann WL, Wu J, Reese T. A mixed methods social network analysis of a cross-border drug network: the Fernando Sanchez Organization (FSO). Trends in Organized Crime. 2020;23(2):154-82.

20. Mainas ED. The analysis of criminal and terrorist organisations as social network structures: a quasi-experimental study. International Journal of Police Science & Management. 2012;14(3):264-82.

21. Malm A, Bichler G. Networks of Collaborating Criminals: Assessing the Structural Vulnerability of Drug Markets. Journal of Research in Crime and Delinquency. 2011;48(2):271-97. doi: 10.1177/0022427810391535.

22. Malm A, Bichler G. Using friends for money: the positional importance of money-launderers in organized crime. Trends in Organized Crime. 2013;16(4):365-81. doi: 10.1007/s12117-013-9205-5.

23. Malm AE, Kinney JB, Pollard NR. Social Network and Distance Correlates of Criminal Associates Involved in Illicit Drug Production. Security Journal. 2008;21(1-2):77-94. doi: 10.1057/palgrave.sj.8350069.

24. Malm A, Bichler G, Van De Walle S. Comparing the ties that bind criminal networks: Is blood thicker than water? Security Journal. 2010;23(1):52-74.

25. Masias VH, Valle M, Morselli C, Crespo F, Vargas A, Laengle S. Modeling Verdict Outcomes Using Social Network Measures: The Watergate and Caviar Network Cases. PLoS One. 2016;11(1):e0147248. Epub 20160129. doi: 10.1371/journal.pone.0147248. PubMed PMID: 26824351; PubMed Central PMCID: PMCPMC4732755.

26. Morselli C. Hells Angels in springtime. Trends in organized crime. 2009;12(2):145-58.

27. Morselli C. Assessing vulnerable and strategic positions in a criminal network. Journal of Contemporary Criminal Justice. 2010;26(4):382-92.

28. Morselli C, Giguere C. Legitimate strengths in criminal networks. Crime, Law and Social Change. 2006;45(3):185-200.

29. Morselli C, Petit K. Law-Enforcement Disruption of a Drug Importation Network. Global Crime. 2007;8(2):109-30. doi: 10.1080/17440570701362208.

30. Morselli C, Giguère C, Petit K. The efficiency/security trade-off in criminal networks. Social networks. 2007;29(1):143-53.

31. Natarajan M. Understanding the structure of a drug trafficking organization: a conversational analysis. Crime Prevention Studies. 2000;11:273-98.

32. Natarajan M. Understanding the Structure of a Large Heroin Distribution Network: A Quantitative Analysis of Qualitative Data. Journal of Quantitative Criminology. 2006;22(2):171-92. doi: 10.1007/s10940-006-9007-x.

33. Norbutas L. Offline constraints in online drug marketplaces: An exploratory analysis of a cryptomarket trade network. Int J Drug Policy. 2018;56:92-100. doi: 10.1016/j.drugpo.2018.03.016.

34. O'Reilly MJA, Hughes CE, Bright DA, Ritter A. Structural and functional changes in an Australian high-level drug trafficking network after exposure to supply changes. Int J Drug Policy. 2020;84:102797. Epub 20200804. doi: 10.1016/j.drugpo.2020.102797. PubMed PMID: 32763755.

35. Roberts RJ. Re-Spatializing Gangs in the United States: An Analysis of Macro-and Micro-Level Network Structures. 2021.

36. Skillicorn DB, Zheng Q, Morselli C. Spectral embedding for dynamic social networks. Proceedings of the 2013 IEEE/ACM International Conference on Advances in Social Networks Analysis and Mining. 2013:316-23.

37. Spear SE. Coordination of care in substance abuse treatment: An interorganizational perspective: UCLA; 2012.

38. Tenti V, Morselli C. Group co-offending networks in Italy’s illegal drug trade. Crime, Law and Social Change. 2014;62(1):21-44. doi: 10.1007/s10611-014-9518-6.

39. Turhal T. Organizational structure of PKK and non-PKK-linked Turkish drug trafficking organizations: The influence of social bonds: George Mason University; 2015.

40. Ünal MC. Do terrorists make a difference in criminal networks? An empirical analysis on illicit drug and narco-terror networks in their prioritization between security and efficiency. Social Networks. 2019;57:1-17. doi: 10.1016/j.socnet.2018.11.001.

41. Wendel T, Khan B, Dombrowski K, Curtis R, McLean K, Misshula E, et al. Dynamics of methamphetamine markets in New York City: Final technical report to the National Institute of Justice. U.S. Department of Justice, 2011.

42. Wood G. The structure and vulnerability of a drug trafficking collaboration network. Social Networks. 2017;48:1-9. doi: 10.1016/j.socnet.2016.07.001.

43. Xu J, Chen H. The topology of dark networks. Communications of the ACM. 2008;51(10):58-65.

44. Xu J, Marshall B, Kaza S, Chen H. Analyzing and visualizing criminal network dynamics: A case study. International Conference on Intelligence and Security Informatics. 2004:359-77.

45. Morselli C, Paquet-Clouston M, Provost C. The independent’s edge in an illegal drug distribution setting: Levitt and Venkatesh revisited. Social Networks. 2017;51:118-26.

46. Xu J, Chen H. Untangling Criminal Networks: A Case Study. In: Chen H, Miranda R, Zeng DD, Demchak C, Schroeder J, Madhusudan T, editors. Intelligence and Security Informatics. Berlin, Heidelberg: Springer Berlin Heidelberg; 2003. p. 232-48.

47. Salazar B, Restrepo LM. Lethal closeness: The evolution of a small-world drug trafficking network. Desafios. 2011;23(2):197-221.

48. Bright D, Koskinen J, Malm A. Illicit network dynamics: The formation and evolution of a drug trafficking network. Journal of Quantitative Criminology. 2019;35(2):237-58.

49. Arimoto MV. Peer Influence and Adolescent Substance Use: A Social Networks Analysis: Washington State University; 2010.

50. Bell DC, Atkinson JS, Carlson JW. Centrality measures for disease transmission networks. Social Networks. 1999;21(1):1-21.

51. Bouchard M, Hashimi S, Tsai K, Lampkin H, Jozaghi E. Back to the core: A network approach to bolster harm reduction among persons who inject drugs. Int J Drug Policy. 2018;51:95-104. doi: 10.1016/j.drugpo.2017.10.006.

52. Dombrowski K, Curtis R, Friedman S, Khan B. Topological and Historical Considerations for Infectious Disease Transmission among Injecting Drug Users in Bushwick, Brooklyn (USA). World J AIDS. 2013;3(1):1-9. doi: 10.4236/wja.2013.31001.

53. Dombrowski K, Khan B, McLean K, Curtis R, Wendel T, Misshula E, et al. A reexamination of connectivity trends via exponential random graph modeling in two IDU risk networks. Substance Use & Misuse. 2013;48(14):1485-97.

54. Friedman SR, Neaigus A, Jose B, Curtis R, Goldstein M, Ildefonso G, et al. Sociometric risk networks and risk for HIV infection. American Journal of Public Health. 1997;87(8):1289-96.

55. Gyarmathy VA, Caplinskiene I, Caplinskas S, Latkin CA. Social network structure and HIV infection among injecting drug users in Lithuania: gatekeepers as bridges of infection. AIDS and Behavior. 2014;18(3):505-10.

56. Heckathorn DD, Broadhead RS, Anthony DL, Weakliem DL. AIDS and social networks: HIV prevention through network mobilization. Sociological Focus. 1999;32(2):159-79.

57. Jonas AB, Young AM, Oser CB, Leukefeld CG, Havens JR. OxyContin(R) as currency: OxyContin(R) use and increased social capital among rural Appalachian drug users. Soc Sci Med. 2012;74(10):1602-9. doi: 10.1016/j.socscimed.2011.12.053.

58. Li J, Weeks MR, Borgatti SP, Clair S, Dickson-Gomez J. A social network approach to demonstrate the diffusion and change process of intervention from peer health advocates to the drug using community. Subst Use Misuse. 2012;47(5):474-90. doi: 10.3109/10826084.2012.644097.

59. Murfree ST. An examination of the social and community context of substance use disorder recovery support services in Rutherford County, Tennessee: Middle Tennessee State University; 2021.

60. Rudolph AE, Young AM, Havens JR. Examining the social context of injection drug use: social proximity to persons who inject drugs versus geographic proximity to persons who inject drugs. American Journal of Epidemiology. 2017;186(8):970-8.

61. Schaefer DR, Davidson KM, Haynie DL, Bouchard M. Network integration within a prison-based therapeutic community. Social Networks. 2021;64:16-28. doi: https://doi.org/10.1016/j.socnet.2020.07.007.

62. Shahesmaeili A, Haghdoost AA, Soori H. Network location and risk of human immunodeficiency virus transmission among injecting drug users: Results of multiple membership multilevel modeling of social networks. Addiction & Health. 2015;7(1-2):1-13.

63. Silva LD, Strobbe S, Oliveira JL, Almeida LY, Cardano M, Souza J. Social support networks of users of crack cocaine and the role of a Brazilian health program for people living on the street: A qualitative study. Arch Psychiatr Nurs. 2021;35(5):526-33. doi: 10.1016/j.apnu.2021.06.010.

64. Singleton AL, Marshall BDL, Bessey S, Harrison MT, Galvani AP, Yedinak JL, et al. Network structure and rapid HIV transmission among people who inject drugs: A simulation-based analysis. Epidemics. 2021;34. doi: 10.1016/j.epidem.2020.100426. PubMed PMID: 33341667.

65. Weeks MR, Clair S, Borgatti SP, Radda K, Schensul JJ. Social networks of drug users in high-risk sites: Finding the connections. AIDS and Behavior. 2002;6(2):193-206.

66. Young A, Jonas A, Mullins U, Halgin DS, Havens J. Network structure and the risk for HIV transmission among rural drug users. AIDS and Behavior. 2013;17(7):2341-51.

67. Young AM, DiClemente RJ, Halgin DS, Sterk CE, Havens JR. Drug users' willingness to encourage social, sexual, and drug network members to receive an HIV vaccine: a social network analysis. AIDS Behav. 2014;18(9):1753-63. doi: 10.1007/s10461-014-0797-9.

68. Young AM, Halgin DS, DiClemente RJ, Sterk CE, Havens JR. Will HIV vaccination reshape HIV risk behavior networks? A social network analysis of drug users' anticipated risk compensation. PLoS One. 2014;9(7):e101047. doi: 10.1371/journal.pone.0101047.

69. Yoon S, Odlum M, Broadwell P, Davis N, Cho H, Deng N, et al. Application of social network analysis of COVID-19 related tweets mentioning cannabis and opioids to gain insights for drug abuse research. Stud Health Technol Inform. 2020;272:5-8. doi: 10.3233/SHTI200479.

70. Koturovic D. Organised networks in Serbia: Crime control and state capture in a country undergoing democratic transition and EU accession: University of Sheffield; 2019.

71. Musto C. Regulating Cannabis Markets. The construction of an innovative drug policy in Uruguay: University of Kent, Utrecht University; 2018.

72. Shomade SA. Case study of the structures of criminal and drug courts: The University of Arizona; 2007.
